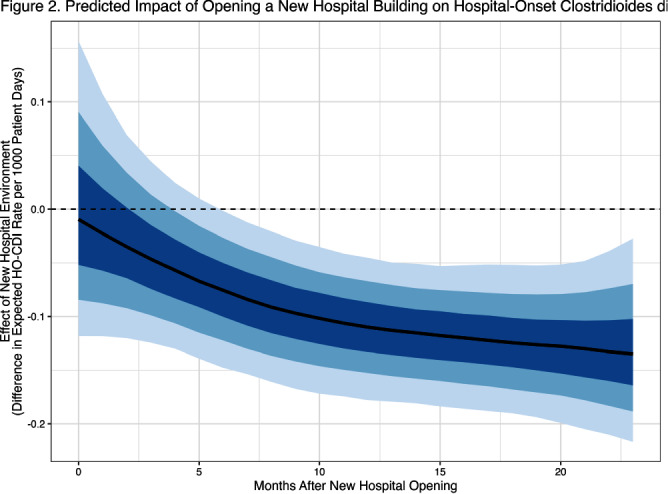# Resetting the environmental reservoir; evaluating the impact of a new hospital building on Clostridioides difficile infection

**DOI:** 10.1017/ash.2024.188

**Published:** 2024-09-16

**Authors:** Liam Giberson, Brendan Kelly, Pam Tolomeo, Leigh Cressman, Laura Cowden, Laurel Glaser, Matthew Ziegler

**Affiliations:** Hospital of the University of Pennsylvania; University of Pennsylvania; University of Pennsylvania Perelman School of Medicine; University of Pennsylvania/Dept. of Biostatistics, Epidemiology and Informatics

## Abstract

**Background:** Prior research has implicated contaminated surfaces in the transmission of Clostridioides difficile within the hospital. To reduce the risk of transmission, enhanced environmental hygiene is performed in rooms of patients with known C.difficile infection (CDI). We wished to evaluate the residual impact of environmental surfaces on hospital-onset CDI (HO-CDI) by comparing HO-CDI rates before and after the opening of a new 504-bed hospital building, HUP Pavilion (PAV). We hypothesized that we would observe a reduction in HO-CDI after opening of PAV due to a reduced burden of C.difficile spores in the environment. **Methods:** We included NHSN reported HO-CDI rates for 28 months prior and 24 months after opening of PAV. Upon opening, patients were divided between the old building (HUP) and PAV. We included all patient units before and after opening. We created hierarchical models of HO-CDI rates using Stan Hamiltonian Monte Carlo (HMC) version 2.30.1, via the “cmdstanr” and “brms” packages with a GAM smooth function by month and intervention period with default, weakly-informative priors. **Results:** At baseline, there was an average of approximately 20,100 patient days per month, subsequently divided between HUP and PAV (mean 10,100 and 12,100 patient days per month). After opening of PAV, we observed a reduced HO-CDI rate (mean 0.21 vs 0.31 per 1000 patient days, P=0.01). When comparing the two specific buildings after opening of PAV, there was a greater reduction noticed in the old building (HUP) as compared to the new building (PAV) (0.12 vs 0.29 per 1000 patient days) (Figure 1). The predicted contrast in HO-CDI rate (Figure 2), shows no immediate change in HO-CDI after opening, however a sustained reduction estimated at 0.1 HO-CDI events per 1000 patient days for the duration of follow-up. **Conclusions:** We observed a reduction in HO-CDI rates after the opening of a new hospital building. The difference in HO-CDI rates between hospital buildings after the move is likely due to the concentration of high-risk patient cohorts within this building. Our findings suggests that there remains an opportunity to reduce HO-CDI through environmental hygiene. However, it is possible that other factors beyond surface environment contributed to an observed reduction in HO-CDI, including other concurrent infection control interventions that focused on smaller populations within the hospital. In future work we will investigate the durability of this observed effect with additional analyses including patient-level risk for HO-CDI.

**Figure:**
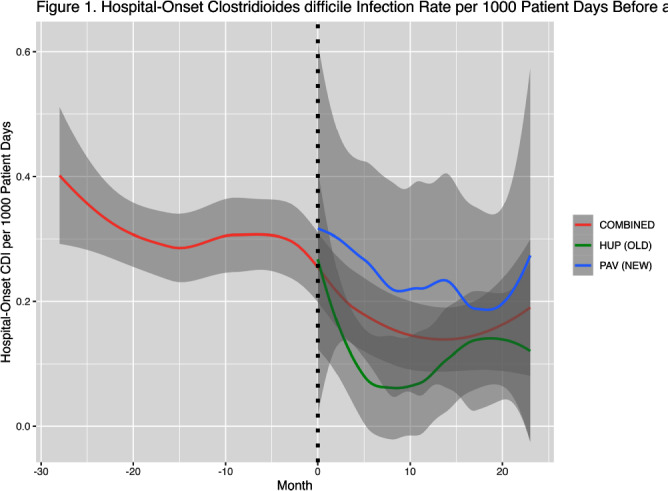

**Figure:**